# Increasing access for biochemistry research in undergraduate education: The malate dehydrogenase CURE community

**DOI:** 10.1016/j.jbc.2022.102298

**Published:** 2022-07-31

**Authors:** Joseph J. Provost

**Affiliations:** Chemistry and Biochemistry, University of San Diego, San Diego, California, USA

**Keywords:** CURE, MCC, MDH, pedagogy, student outcomes, STEM, underrepresented, inquiry, CURE, course-based undergraduate research experience, MDH, malate dehydrogenase, MSI, minority-serving institution, PUI, primarily undergraduate institution, NSF, National Science Foundation, STEM, Science, Technology, Engineering, and Mathematics, URE, undergraduate research experience

## Abstract

Integrating research into the classroom environment is an influential pedagogical tool to support student learning, increase retention of STEM students, and help students identify as scientists. The evolution of course-based undergraduate research experiences (CUREs) has grown from individual faculty incorporating their research in the teaching laboratory into well-supported systems to sustain faculty engagement in CUREs. To support the growth of protein-centric biochemistry-related CUREs, we cultivated a community of enthusiastic faculty to develop and adopt malate dehydrogenase (MDH) as a CURE focal point. The MDH CURE Community has grown into a vibrant and exciting group of over 28 faculty from various institutions, including community colleges, minority-serving institutions, undergraduate institutions, and research-intensive institutions in just 4 years. This collective has also addressed important pedagogical questions on the impact of CURE collaboration and the length of the CURE experience in community colleges, undergraduate institutions, and research-intensive institutions. This work provided evidence that modular or partial-semester CUREs also support student outcomes, especially the positive impact it had on underrepresented students. We are currently focused on expanding the MDH CURE Community network by generating more teaching and research materials, creating regional hubs for local interaction and increasing mentoring capacity, and offering mentoring and professional development opportunities for new faculty adopters.

At a scientist’s core is the innate drive to understand unknown phenomena. As graduate students and postdoctoral trainees, we have been taught to read literature, look for gaps in knowledge, construct hypotheses, and design exciting and powerful experiments to test the central premise. As faculty, we look for students to have that same passion for working “at the bench.” However, reading about science is not the same as doing science. The standard drill and fill laboratory exercises where students perform standard well work experiment and fill in the worksheet no more expose students to the discovery of being a scientist than reading about being a concert pianist enables someone to become a musician. The best way to ignite that drive for discovery is to invest in undergraduates to give them this necessary experience. Traditionally this has been a small group mentored experience in the apprentice model. The undergraduate research experience significantly impacts the motivation and persistence of science students (1, 2, 3, 4, 5) http://rescorp.org/news/2018/06/expanding-the-cure-model-course-based-undergraduate-research-experience (accessed March 11,2022). Providing students with a meaningful and engaging research experience is a high-impact practice. The experience provides an “achievement of deep learning, significant engagement gains, and positive differential impact on historically underserved student populations” (6, 7). Thus, as many students as possible should be given such a valuable research experience.

The logistical strain on large or small schools to provide a research experience to all students is restricting. While research-intensive universities have a deep commitment to advancing science through research, a large student population strains their ability to provide small-group research experiences. Likewise, smaller institutions will have the same issues providing research experiences with fewer faculty and often less resources. At the same time, those institutions whose faculty have a high teaching load restrict the time and resources available to conduct research. Thus, all universities are faced with overwhelming obstacles to creating research opportunities for all students. Summer internships and other research experiences are helpful, but the capacity is limited. Further complicating accessibility is that some students need to work to financially support their families in the summer. In addition, access to research internships often occurs late in a student's college experience, reducing the impact on a student's educational trajectory. Dr Terry Woodin, longtime supporter of education reform and National Science Foundation Program Director, wrote of the importance of widely providing an undergraduate research experience and called to integrate scientific research experiences throughout the curriculum (8, 9). One of the early approaches to engage students in undergraduate teaching laboratories was inquiry-based science (10, 11). Laboratories that utilize known and predictable experimental outcomes provide students with a research-like experience in an inquiry-based laboratory but are limited in the potential impact on student outcomes and do not meet the Council on Undergraduate Research definition of undergraduate research (“A mentored investigation or creative inquiry conducted by undergraduates that seeks to make a scholarly or artistic contribution to knowledge”). Integrating actual research with an unknown experimental outcome is more difficult to achieve in a teaching environment. Inquiry-based science involves an investigative approach to teaching. Students are provided an opportunity to investigate and experiment with a problem where they do not know and are not given a correct answer. Instead of directing students to an established method where students provide a result (think standards and unknowns), instructors challenge students to decide on an experimental design based on observations and open-ended questions. Faculty members are facilitators as students make critical decisions and predictions in the design and execution of their "project." In this case, the instructor knows the assignment's answer, leaving it for the student to find the final results, emphasizing the path of learning over getting a particular value. This approach to integrating a meaningful research experience in the classroom is now labeled a Course-Based Undergraduate Research Experience (CURE). Several extensive reviews of biochemical-related CUREs describe the evolution and many approaches to CUREs in molecular life science (12, 13, 14, 15).

## Why CUREs

The need to support student retention in Science, Technology, Engineering, and Mathematics (STEM) has been a long-standing concern. The National Science Foundation (NSF) reports a 33% retention rate for students in STEM undergraduate degrees, yet predicts a 20.4% increase in employment opportunities in the biological and environmental life sciences and a 12.75% increase in the physical sciences https://www.pewsocialtrends.org/2018/01/09/diversity-in-the-stem-workforce-varies-widely-across-jobs/ (accessed March 12, 2022) (16). A recent study reports that employers in STEM fields expect to have 3.5 million in STEM jobs in the United States by 2025 with a predicted labor shortage in excess of 2 million workers https://www.nsf.gov/statistics/2017/nsf17310/ (accessed Dec 4, 2020). The US Education Department indicates that, of those who started in a STEM degree program, only 52% earned a STEM degree. Moreover, this attrition was worse for those in community college programs, where only 30% of those starting a STEM major earned a degree in a STEM field https://www.nsf.gov/statistics/2017/nsf17310/ (accessed Dec 4, 2020), https://www.nsf.gov/statistics/2018/nsb20181/ (accessed March 12, 2022). Such analysis leads to a national concern about meeting the demand for trained workers in STEM https://www.pewsocialtrends.org/2018/01/09/diversity-in-the-stem-workforce-varies-widely-across-jobs/ (accessed March 12, 2022) (17). Current estimates of undergraduate majors in the physical and life sciences are that white students make up 67 to 68%, Asian-American students represent 16 to 19%, leaving 13 to 15% from Hispanic, African-American, and all other communities (18). The disparity between demographics and representation is even more significant in STEM employment, where Hispanics and African-Americans account for only 6% and 5%, respectively, of science and engineering occupations https://www.pewsocialtrends.org/2018/01/09/diversity-in-the-stem-workforce-varies-widely-across-jobs/ (accessed March 12, 2022) (18). Clearly, there is a need for initiatives that increase the retention of students in STEM fields to support the workforce's needs and help these students achieve their personal goals. Broader access to undergraduate research can help to serve this purpose.

In 1997, I began incorporating research into a year-long biochemistry laboratory that evolved into a formalized experience using a His-tagged GFP fusion protein with malate dehydrogenase (NSF DUE 0088654 and DUE 0511629, 23, 24). The first semester was more traditional, easing into an inquiry where students were guided to make their expression, purification, and characterization choices. In the second semester, students used these skills to fully experience a research-like environment. Students were given a list of wildtype and mutant His-tagged malate dehydrogenase clones, guided through a series of bioinformatics and structural biochemistry workshops, and then asked to generate a testable hypothesis using MDH. For the remainder of the semester, student groups designed and worked on their research project. Assessment of traditional learning objectives showed that students certainly learned traditional laboratory skills. What was critical is that the course had a high retention rate (98% over 3 years) of primarily first-generation and low-income students. In addition, assessment of students’ interest and future plans helped motivate the students to become a scientist experiencing after research in the classroom *versus* those in the control group who experienced traditional laboratories (19). When assessing students' confidence and scientific interest before the course, there was a significant difference in students who had already experienced some level of mentored undergraduate research. However, after a semester of guided inquiry and the second semester of mentored research, this gap significantly decreased (20). Using several assessment tools, students' critical thinking skills were found to have increased over those who did not perform research. Students who continued a traditional research experience were primed and ready to dive deep into the literature, plan experiments, and analyze their data (19, 20). Anecdotally, teaching a laboratory in this manner was a richer experience, more similar to that of a research laboratory filled with interested students. Overall, the benefits to students were measurable and importantly accessible to any student in a biochemistry major.

## What is a CURE?

Several influential faculty, including Drs Erin Dolan, Graham Hatfull, Sarah Elgin, David Lopatto, Sara Brownell, Kim Tanner, and Ellis Bell, have championed the formal integration of research into the classroom environment. Driven to increase access to a critical research experience, faculty have evolved from teaching in an inquiry mode (a project-based course where the outcome is known by the instructor but not the student) to one providing students with a more realistic research opportunity through a Course-Based Undergraduate Research Experience. The simplest definition of a CURE is integrating research into a course where neither the instructor nor the students know the outcome. Essentially, a CURE creates the excitement of conducting research in a classroom laboratory setting. The critical components of a CURE have evolved in its definition over time: from communication, reading literature, and ownership (21) to design, logistics, motivation and student support (22, 23) and now, engaging students; the generation of novel information of significant relevance to students; an engaging in reflection (22) and even collaboration; longitudinal focus on one set of questions over the course; and presentations (24). An early publication by the Dolan group defining the important components of a CURE has been broadly adopted in the biochemistry, molecular biology, and molecular life science community (25). The current defining characteristics of a CURE include five key dimensions that form both design features and support the framework for a logic model to measure the mechanism of effective learning and outcomes of a CURE (25). These features include the use of science practices (designing experiments, hypothesis creation, analyzing data), discovery (research project that is unknown with ownership of student and instructor), relevance (something that is unknown and is essential to a broader audience), collaboration (both in and outside of the classroom), and iteration (students are allowed to fail and repeat experiments). While the description has changed over time, the pedagogical implementation, the impact of CUREs, and the causal mechanism of learning (establishing the process in which a variable/dimension that gives rise to student learning and outcomes) are still being intensely studied.

Implementation of a CURE requires creative and, at times, intensive work. Thus, it is natural to ask why change from a well-established and functional teaching laboratory manual to the unknown of a research project. The simple reason is to increase retention in STEM students as the research experience is expanded beyond the apprentice mode. This creates something exciting and engaging for the students, and in the classroom creates the very reason we have become scientists—an opportunity to do research. The impact of research on student learning reconstitutes in-part the experience of working in a mentored research laboratory as an apprentice, now defined as an undergraduate research experience (URE). The influence on undergraduates involved in UREs has been studied with the most significant impact on student's motivation and persistence (15, 26, 27) http://sites.nationalacademies.org/cs/groups/dbassesite/documents/webpage/dbasse_177288.pdf (Accessed March 12, 2022). The benefits of integrating research for undergraduates into courses were similar to those seen with UREs (15) http://sites.nationalacademies.org/cs/groups/dbassesite/documents/webpage/dbasse_177288.pdf (Accessed March 12, 2022). More specifically, the involvement of undergraduates in research promotes how students think and act as scientists, bolsters their feelings of belonging, and improves their confidence in being a scientist (1, 2, 3). A longitudinal study of over 2000 students as freshmen and again as seniors show that students involved in a URE are more likely to plan to pursue graduate or professional degrees in STEM by 14 to 17% (27). Another reason for using a CURE is that the students learn what we hope they learn about the practice of science. CUREs help a student realize an increased interest in, and motivation for, continued work in science and also an increase in cognitive gains, especially for learning the scientific process (28, 29). While more work is being done to understand the causal mechanism for CURE and URE outcomes, the investment in creating a CURE has a positive impact on students.

There are barriers to creating and implementing a CURE. Several studies point to concerns about the time and effort required to create a new CURE laboratory (29, 30, 31, 32). Others are concerned about the push-back from entrenched faculty and institutions that are slow to adopt change. Students are initially concerned about the level of work, and the uncertainty of an unknown result and complaints can be damaging if faculty are not supported. One way to overcome the effect of single faculty creating and sustaining a CURE is to shift from operating as an individual to becoming part of a mentored and supported community. Larger projects, like SEA-PHAGE and other larger CURE communities, have successfully offset these problems by providing a community to share resources, provide faculty guidance, and enhance communication between adopting instructors. As individual, stand-alone CUREs, faculty are silos working independently of others to create a sustainable collaborative project. Lopatto *et al*. (30) emphasized there is a real need to create collaborative communities of faculty involved in CURE work. This community can create a sustained, long-term experience for the students and decrease the barriers to start integrating research in the teaching laboratory. A community of faculty can create a culture of collaboration to help foster the success of its members. The most successful and sustained CUREs involve a community of faculty creating, evolving, and collaborating on CUREs. There are several examples of established national-level CUREs that support their adoption at various institutions. Three of the most established programs are the HHMI funded Science Education Alliance–Phage Hunters (SEA-PHAGES; www.seaphages.org) targeting first-year life science students, The Genome Consortium for Active Teaching (GCAT; www.bio.davidson.edu/gcat), and Genomic Education Partnership (GEP; www.gep.wustl.edu). These are highly successful because of the inclusive and rich resources supporting faculty engaging in CUREs. Each has a prescribed approach and a shared scientific theme. Faculty involved in these national-level CUREs engage in training workshops, have access to shared teaching materials, and thus create a clear roadmap to readily adopt and reduce the activation barrier to starting a CURE. While the benefits to these large consortiums are many, the constant challenge such collaborative networks help solve is sustainability. Nevertheless, most CUREs are individual efforts conducted by isolated faculty. Serc.carleton has an established network to share their programs and impressively support their overall efforts (32). However, faculty have sparse opportunities to create a protein-centric, biochemistry CURE as most supportive systems are organismal or molecular biology and genetics in nature. Only two groups have addressed this need: the NSF-funded BASIL program, which has established modules and protocols for conducting inquiry-based research using 4000 unknown proteins (BASIL; www.basilbiochem.github.io/basil/index.html, (33, 34)), and our Malate Dehydrogenase CURE Community (MCC; https://mdh-cures-community.squarespace.com, (12)).

## MDH CUREs

The Malate Dehydrogenase CUREs Community is a collaborative community of faculty focused on developing protein-centric CUREs for a broad spectrum of institutions. Started in 2017 with 11 institutions including two community colleges and two minority-serving institutions (MSIs), the group was designed to address two major pedagogical questions about effective aspects of CUREs. These were 1) the impact of length of CURE project, specifically if a modular CURE (5–6 weeks) incorporated within a laboratory course would be as effective as an entire course CURE experience, and 2) the effect of collaboration, specifically if interinstitutional collaboration would be more effective than CUREs involving a single institution. Since then, the community has grown to 30 institutions with increases in both community colleges and MSIs. In addition to the pedagogical questions examined, the MCC project engaged faculty from a diverse array of institution types to develop and evaluate resources needed to support MDH protein-centric CURE. The MCC project provides guidelines to design and execute an MDH CURE, a collection of best practices that successfully manage semester-long or modular CUREs and experimental protocols. It also provides a critical component of instructor support, including the opportunity to collaborate scientifically on the technical aspects of teaching a CURE. In response to the COVID pandemic, faculty in the MCC rapidly developed computational approaches to allow CURE courses to continue in a remote format without sacrificing the components that make CUREs effective.

To catalyze the creation of a community and foster these essential shared faculty interactions, the MCC (NSF DUE-1726932 PI J.E. Bell CoPIs J.J. Provost and J.K. Bell) focuses on the enzyme malate dehydrogenase (MDH). MDH is stable, inexpensive to assay, and purified easily in undergraduate laboratories utilizing a histidine tag. In addition, using MDH by all groups provides a common focus for a research community, facilitating the incorporation of scientific collaborations between institutions that we have demonstrated increases student learning.

**Figure 1:**
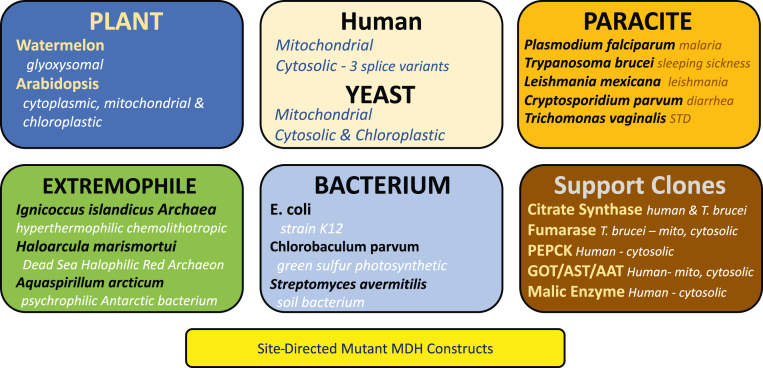
**Available MDH isoforms in bacterial expression vectors for CURE adoption**.

**Figure 2:**
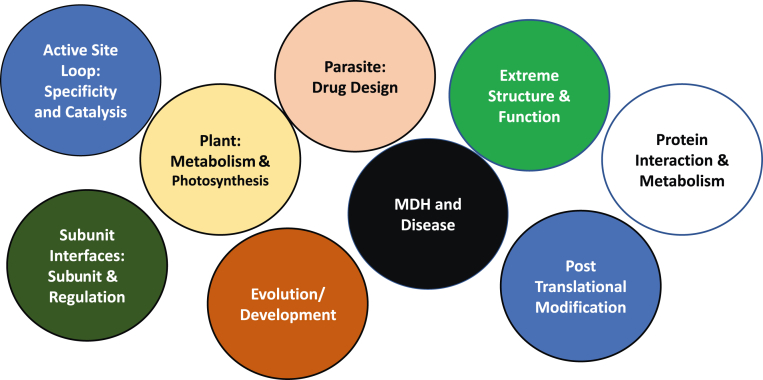
**MDH CURE potential project areas**.

To support MDH CUREs, the MCC generated 22 bacterially expressed and wildtype MDH His-tagged constructs from a wide range of MDH isoforms (Fig. 1). As a result, adopting faculty can now select MDH from distinct evolutionary organisms for structurally or enzymatically interesting scientific questions (Fig. 2). In addition to the wildtype MDH clones, students engaged in MCC CUREs generated more than 100 site-directed mutants to study a variety of protein structure–function relationships. These clones were sequence verified and made available for MDH CURE adopters. Each isoform has been checked for expression and base-level activity under standard conditions, and stability and storage studies have been performed.

With these resources, faculty can ask a vast range of biochemical and physiological questions using MDH in potential future CURE projects. For example, human cytosolic MDH alone has three splice forms for which almost no kinetic or regulatory information exists. Likewise, mass spectroscopy detected 18 potential functional phosphorylation sites on human mitochondrial MDH, yet the impact on MDH behavior has not been studied. Interactions between MDH and other cytosolic and mitochondrial proteins have been studied on a limited basis in plants, mammalian cells, and some parasitic organisms, but many others are possible.

Central to teaching CUREs in the MCC is to engage students in hypothesis development. Bell *et. al*. outline this key dimensional activity in a mini-curricula guide for adopting faculty for students at all levels from entry-level or gateway courses to the upper-division course for science majors (35). Students are exposed to the importance of designing hypothesis-driven experiments *versus* seeing hypothesis development as a “guess.” This helps students better connect the current literature with an important scientific question. Teaching students how to generate a hypothesis should contain, at minimum reading the literature to identify the big picture, evaluating preliminary evidence or conducting bioinformatic/structural analysis to help define the scientific problem, creating a specific hypothesis from this scientific question, and making predictions and designing experiments from these elements.

### Impact of the MCC

As part of the MCC, we have asked critical questions on implementing and tailoring CUREs for individual institutions. When the role of CUREs on underrepresented students was measured, Zhang, J. *et al*. (36) report that, while all student groups increased their desire to engage in research after the CURE experience, the impact of CUREs on underrepresented students at community colleges was greater than other groups (who also increased their plans to engage in research post CURE) (36) This same study found that, while MDH CUREs were successful in improving the scientific aptitude of students in research-intensive institutions, the effect was more significant in both community colleges and primarily undergraduate institutions (PUIs). Interestingly, the impact was greatest on the undergraduate students at community colleges. This group showed the most significant gains in metrics evaluating their ability to design experiments and in their propensity to continue in science. As suggested by the authors, the implementation of CUREs can help reduce the gap for the next generation of life scientists. Another study by the MCC faculty found that underrepresented students showed significantly higher interest in research in the future over other groups even when they participated in a shortened, half-semester CURE (37). This breakout of CURE's impact on student groups has not been shown before and supports CUREs to help improve retention of this population of students in STEM. A second focus of the pedagogical research on the MCC project was to investigate the role of collaboration between institutions involved in MDH CUREs in student outcomes. Here the authors examined the effect of involving faculty from outside institutions on student gains in experimental design and on their attitudes of their skills, and the importance of STEM (data not shown, manuscript in preparation). The benefits of an outside faculty visiting (*via* zoom or other approaches) to discuss their science, share data, and conduct the types of collaborations found in modern research laboratories were increased compared with students conducting CUREs in their institution. The authors of this work shared how faculty involved in collaborative CUREs reported the authentic excitement by the students and that having another outside scientist visit with them increased their motivation for their project. Moreover, faculty involved with the MCC, regardless of the type of CURE they conducted, report that having another group to share experiences, ask for troubleshooting advice, and discuss the logistics and management of a shared project were immensely beneficial. This observation rang true for nearly all faculty involved, regardless of the institution type or student population.

What next? The next phase to support molecular life scientists who wish to integrate an MDH CURE in their classroom is to expand the network, engage new faculty in workshops, and expand the resources available to reduce the barriers to adoption. The mission of the MCC has evolved from creating the framework of novel protein-centric CUREs and testing educational hypotheses to becoming an inclusive and sustainable community consisting of a national network of faculty organized into three regional hubs. Faculty currently involved in MDH CUREs are from various institutions, including community colleges, MSIs (including Historically Black Colleges and Universities and Hispanic Serving Institutions), PUIs, and research-intensive institutions. Funded by an NSF RCN UBE award (DUE 2119918, P. I./CoPIs J. J. Provost, A. Springer, L. Gentile, and J. E. Bell), the MCC's goal is to increase the number of students from a broad range of backgrounds to experience the positive results of conducting research. To achieve these goals, the MCC has several new foci of activity. The first is to increase the online support for new and current MDH CURE adopters. The MCC community provided input to what was important and helpful in starting and sustaining MDH CUREs. The MCC website (https://mdh-cures-community.squarespace.com) is a robust and growing resource for any faculty interested in the MCC. Activities include interactive and dynamic support for faculty needing guidance on applying a CURE, resources for syllabi, and other teaching material to make entry into the MCC easier and more student focused. All MDH clones and other proteins involved in MDH interactions have resource files and protocols for experiments in a potential CURE. The MCC is partnering with Addgene and Bio-Rad to provide easy and long-term access to plasmids and teaching materials for those wishing to adopt an MDH CURE. The MCC is organized into three regional hubs to provide hands-on workshops and personal mentoring. Each hub's mission is to support faculty adoption with small regional workshops that cover critical issues in adopting a CURE, discuss the challenges at different institutions, disseminate results, and create a network of MDH experienced faculty ready to mentor the next generation of MCC faculty. The MCC now recognizes the growing cadre of experienced MDH CURE faculty who are ready to mentor and support new faculty as MCC Faculty Fellows. New faculty or instructors (including adjuncts, laboratory directors, and postdoctoral fellows) interested in developing an MDH CURE and becoming part of the national group of supportive educators working together to provide this research opportunity can be connected with an MCC Faculty Fellow. The MCC will also host a unique training opportunity to support a new cohort of faculty who wish to teach a CURE-format laboratory based on MDH. Each year we will host a MCC Faculty Fellows Cohort group to focus on faculty from underresourced institutions and those working with large populations of students from backgrounds traditionally underrepresented in higher education. Each Cohort of Faculty Fellows will include faculty from various institutions, including community colleges, MSIs, PUIs, and regional comprehensive and research-intensive universities. They will have different types of teaching responsibilities, including introductory, advanced, interdisciplinary, remote/online, and will be at different stages in their careers (new faculty members, experienced faculty members interested in changing pedagogical approaches). The MCC Faculty Fellows Cohort will be provided with funding for 2 years of professional development, starting with a combination of a virtual and in-person workshop on developing an MDH CURE and will be provided with mentoring throughout the process.

## Conclusion

Regardless of the kind of research we conduct, the excitement of science is a powerful tool that should be integrated into all undergraduate experiences. The impact of exposures to authentic and meaningful science is well documented and helps retain students, which will support a vibrant and diverse workforce in molecular life sciences in the future. Therefore, we have created a novel supported community to engage students in CUREs and are in the process of expanding this network to ensure more students from all institutions at all levels (first year to senior year) can experience this vital activity.

## Conflict of interest

The authors declare that they have no conflicts of interest with the contents of this article.
